# CTRP12 in cardiovascular and metabolic diseases: current status and future perspective

**DOI:** 10.3389/fcvm.2026.1749963

**Published:** 2026-02-04

**Authors:** Huan Wan, Zi-Zhen Zhang, Xiao-Hua Yu, Dan-Ni Liu, Ji Min, Jin Zou

**Affiliations:** 1 The First Affiliated Hospital, Department of Cardiology, Hengyang Medical School, University of South China, Hengyang, Hunan, China; 2 School of Medicine, Hunan Polytechnic of Environment and Biology, Hengyang, Hunan, China; 3 Institute of Clinical Medicine, The Second Affiliated Hospital of Hainan Medical University, Haikou, Hainan, China

**Keywords:** coronary artery disease, CTRP12, diabetes, heart failure, obesity

## Abstract

Cardiovascular and metabolic diseases severely threat human health with their management being a global challenge. C1q tumor necrosis factor-related protein 12 (CTRP12), a newly identified adipokine and paralog of adiponectin, is expressed predominantly by adipose tissue. As a secretory protein, full-length CTRP12 can be cleaved by furin to produce a globular isoform, which is the major form in the blood stream. CTRP12 has anti-inflammatory and glucose-lowering properties and also participates in the regulation of lipid metabolism, oxidative stress and cell apoptosis. Dysregulation of CTRP12 has been shown be involved in the occurrence and development of cardiovascular and metabolic diseases, including coronary artery disease, heart failure, diabetes, obesity and non-alcoholic fatty liver disease. Thus, targeting CTRP12 may have important implications in preventing and treating cardiovascular and metabolic diseases. To provide a comprehensive understanding of the role of CTRP12 in cardiovascular and metabolic diseases, this review delved into the structural characteristics and biological functions with an emphasis on its link to several major cardiovascular and metabolic diseases.

## Highlights

CTRP12 is a newly discovered adipokine with multiple biological functions.
CTRP12 plays an important role in the development of CVMDs.
CTRP12 could be developed as a drug target for the treatment of CVMDs.

## 1 Introduction

Cardiovascular diseases (CVDs) are a group of disorders impairing heart and blood vessels, predominantly encompassing coronary artery disease (CAD), cerebrovascular disease, and peripheral arterial disease. The pathogenesis of CVDs is complex and involves numerous pathological factors and signaling pathways, such as lipid metabolism dysregulation, inflammatory response and cell death (1–3). Although statin treatment has been widely used to modulate blood lipid levels, patients with established CVDs are often left with residual cardiovascular risk (4). Metabolic diseases are a type of disorders that primarily impede glucose and lipid metabolism, consisting of diabetes, obesity and non-alcoholic fatty liver disease (NAFLD). With aging and changes of life style, the morbidity and mortality of cardiovascular and metabolic diseases continues to increase in recent years, bringing a huge economic burden across the world (5–7). Prevention and treatment of cardiovascular and metabolic diseases have become a major challenge despite great progress has been made in this field. Thus, a comprehensive understanding of the role of key players in cardiovascular and metabolic diseases is of great scientific significance and clinical value.

Adipose tissue not only plays a critical role in energy storage but also functions as a large active endocrine organ that can express and secrete a variety of adipokines, including adiponectin, leptin, visfatin, apelin and C1q tumor necrosis factor-related proteins (CTRPs). As the most extensively investigated adipokine, adiponectin is essential to maintain normal cardiovascular and metabolic functions (8, 9). CTRPs belong to a highly conserved family of adiponectin paralogs and share significant sequence homology with the immune complement component C1q (10). Currently, 15 members have been identified, ranging from CTRP1 to CTRP15. CTRP12, also called adipolin, is a recently discovered adipokine and plays an important role in regulating cardiovascular and metabolic functions (11). Dysregulation of CTRP12 has been shown to be closely associated with cardiovascular and metabolic diseases, such as CAD, heart failure, diabetes, obesity and NAFLD (12, 13). This sheds lights on CTRP12 as a novel and promising target for the prevention and treatment of cardiovascular and metabolic diseases. In this review, we summarized the current knowledge about the role of CTRP12 in cardiovascular and metabolic diseases to provide an important framework for future investigation and therapeutic intervention.

## 2 The origin, structure, metabolic pathway and biological properties of CTRP12

**Figure 1:**
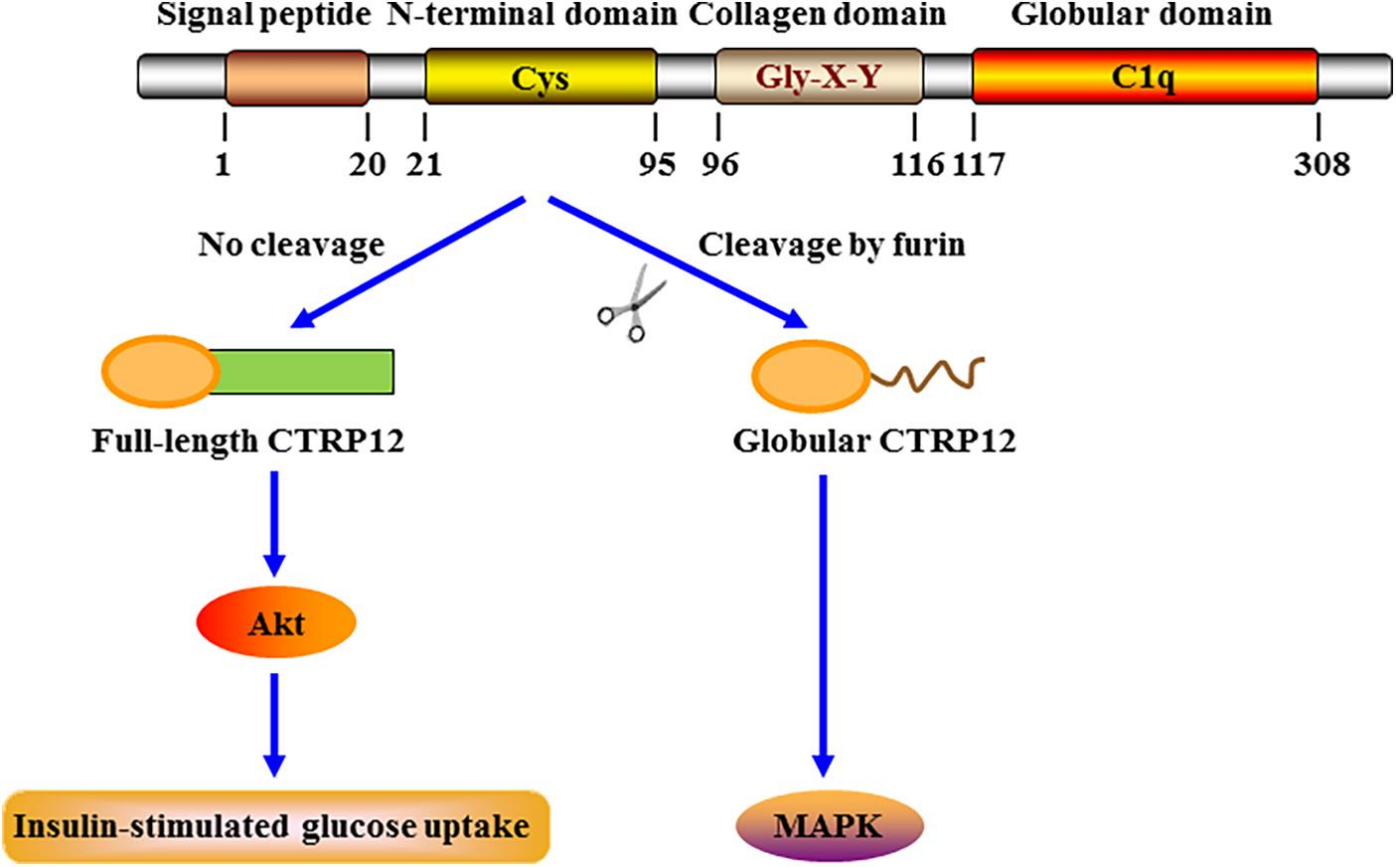
Schematic structure, cleavage and signaling specificity of CTRP12. CTRP12 is made up of four domains: a signal peptide for secretion, an N-terminal domain with multiple conserved Cys residues, a collagen domain with Gly-X-Y repeats, and a C-terminal globular domain homologous to the immune complement C1q. The full-length CTRP12 protein can be cleaved by furin to generate a globular isoform as the predominant form in circulation. Full-length CTRP12 preferentially activates Akt signaling to facilitate insulin-stimulated glucose uptake. Globular CTRP12 preferentially activates MAPK signaling.

CTRP12 was first identified as an insulin-sensitizing adipokine by Enomoto et al. in 2011 (14). Mouse *CTRP12* gene (Gene ID: 67389) contains 8 exons and is mapped to chromosome 4 E2. Like adiponectin, mouse CTRP12 protein is divided into 4 regions: a signal peptide that can direct protein secretion, an N-terminal domain essential for the assembly of higher order oligomeric structure, a collagen domain with six Gly-X-Y repeats, and a C-terminal globular C1q domain with three conserved Cys residues and two potential *N*-glycosylation sites (Figure 1). The N-terminal domain contains three potential *N*-linked glycosylation sites, four Cys residues and an endopeptidase cleavage motif (KKXR). These features indicate that the protein likely undergoes multiple types of posttranslational modification. Adiponectin is known to be expressed almost exclusively by adipocytes (15). However, CTRP12 is more widely expressed in mice, with the comparable expression levels in white and brown adipose tissues (16).

Human *CTRP12* gene (Gene ID: 388581) encompasses 9 exons and is localized in chromosome 1p36.33. Human CTRP12 protein and its corresponding mouse ortholog share 71% and 83% amino acid identity in their full-length form and globular domain, respectively (11). Similar to adiponectin, CTRP12 is expressed predominantly by adipose tissue in humans, with high induction during adipogenesis. Moreover, the higher expression of human CTRP12 mRNA in adipose tissue is correlated with its higher serum levels (16). CTRP12 is highly conserved throughout vertebrate evolution, including dogs, chickens, zebrafishes, frogs, mice and humans. Dysregulation of CTRP12 expression has been frequently observed in a variety of diseases. For instance, increased CTRP12 expression is seen during chondrocyte differentiation, suggesting that this adipokine may be involved in cartilage maturation and development (17). Serum CTRP12 levels in Hashimoto's thyroiditis patients are higher than those in healthy subjects (18). In patients with chronic obstructive pulmonary disease, serum CTRP12 levels are decreased as compared to healthy individuals and show a negative correlation with disease severity (19). Although no statistically significant difference is determined between endometrial cancer patients with and without omentectomy in respect of serum CTRP12 levels measured preoperatively, the postoperative blood levels of CTRP12 are significantly increased compared with the preoperative value (20). This reveals a positive effect of omentectomy on CTRP12 metabolism in endometrial cancer patients.

Krueppel-like factor 15 (KLF15) is a significant member of the zinc-finger family of transcription factors. It has been reported that KLF15 is able to up-regulate the expression of CTRP12 by binding to its promoter in adipocytes (21). KLF3 is another transcription factor involving the modulation of adipogenesis (22). Bell-Anderson et al. found that KLF3 inhibits CTRP12 transcription and consequently leads to glucose intolerance in mice (23). In subcutaneous adipose tissue explants, insulin dramatically enhances the expression and secretion of CTRP12 by activating phosphatidylinositol 3-kinase (24). Metformin belongs to one of first-line therapeutic drugs for type 2 diabetes mellitus. Treatment with metformin leads to a significant increase in CTRP12 protein levels in subcutaneous adipose tissue, and this effect is attenuated by the inhibitor of AMP-activated protein kinase (25).

CTRP12 circulates in plasma as a full-length protein or a cleaved globular isoform, with the latter as the major circulating isoform (Figure 1). Furin, also called proprotein convertase subtilisin/kexin 3, is an enzyme that belongs to the subtilisin-like proprotein convertase family. It has been demonstrated that furin is able to mediate the cleavage of CTRP12 at Lys-91 within the N-terminal KKXR motif (26). Treatment of adipocytes with tumor necrosis factor-α (TNF-α) dramatically up-regulates furin expression, leading to increased production of the cleaved form (27). After secretion from mammalian cells, CTRP12 is subjected to *N*-linked glycosylation. Three *N*-linked glycosylation sites have been identified, designated as Asn-39, Asn-287, and Asn-297. When Asn-39 and Asn-297 are glycosylated, the cleavage of CTRP12 by furin is inhibited; however, Asn-287 is required for proper protein folding of CTRP12, which is independent of its glycosylation (28). It is important to note that both isoforms differ in oligomeric structure and signal transduction (26). The full-length CTRP12 protein forms the trimers and larger complexes and has stronger effects on the activation of Akt signaling. In contrast, the cleaved globular isoform of CTRP12 predominantly forms the dimers and preferentially activates mitogen-activated protein kinase (MAPK) signaling. Moreover, only full-length CTRP12 protein can promote glucose uptake stimulated by insulin in adipocytes. Thus, enhancing the proportion of full-length CTRP12 protein could be a more effective approach to improve insulin sensitivity.

## 3 Physiological function and pathological role of CTRP12

**Figure 2:**
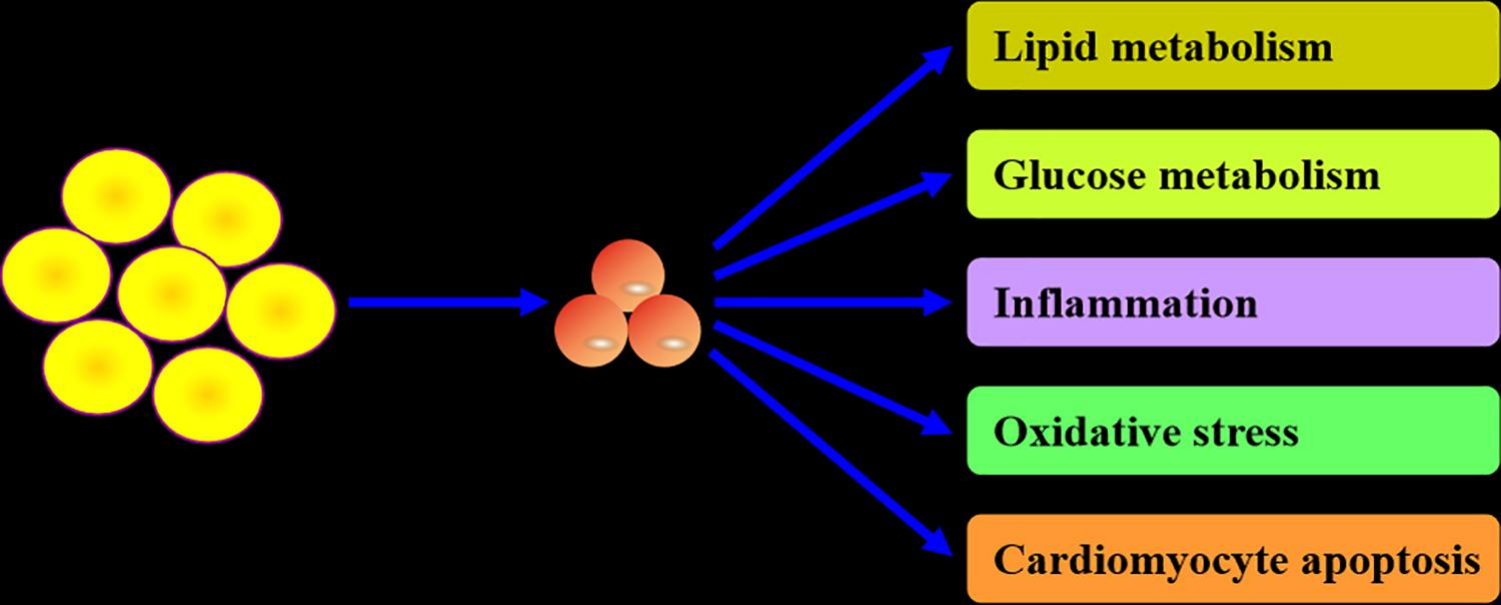
Biological actions of CTRP12. CTRP12 is produced predominantly by adipocytes. As a multifunctional adipokine, CTRP12 participates in the regulation of lipid and glucose metabolism, inflammation, oxidative stress and cardiomyocyte apoptosis.

As a secretory protein, CTRP12 plays an important role in regulating lipid and glucose metabolism, inflammatory response, oxidative stress and cardiomyocyte apoptosis (Figure 2). These contents are discussed individually below.

### 3.1 Lipid metabolism

Lipids predominantly contain fatty acids, cholesterol, phospholipids and triglycerides. They not only constitute the basic composition of plasma membrane but also play a crucial role in energy storage and signal transduction. Aberrant lipid metabolism is associated with a variety of diseases such as obesity and atherosclerosis (29). It is suggested that circulating CTRP12 levels show a positive correlation with high-density lipoprotein cholesterol (HDL-C) in CAD patients (30). Recent work from our group revealed that overexpression of CTRP12 inhibits macrophage lipid accumulation by stimulating cholesterol efflux (31). In hepatoma cells and primary mouse hepatocytes, treatment with recombinant CTRP12 protein inhibits lipogenesis by down-regulating the expression of glycerophosphate acyltransferase (GPAT) and diacylglycerol acyltransferase (DGAT), two key enzymes involving triglyceride synthesis (32). CTRP12 also reduces the export of very low-density lipoprotein-triglycerides from hepatocytes by suppressing the hepatocyte nuclear factor 4A (HNF4A)/microsomal triglyceride transfer protein (MTTP) signaling pathway (32). When challenged with a high-fat diet, CTRP12^+/−^ male mice exhibit decreased triglyceride secretion and increased triglyceride and cholesterol levels in liver tissue (33). In contrast to male mice, partial deficiency of CTRP12 promotes hepatic triglyceride secretion in female mice fed a high-fat diet (33). These results suggest that loss of a single *CTRP12* allele regulates lipid metabolism in a sex-dependent manner, highlighting the importance of genetic determinant in metabolic phenotypes. Of note, female mice exhibit higher levels of CTRP9 transcripts when compared with male mice, revealing a gender-biased expression pattern (34). Nevertheless, whether CTRP12 expression differs between males and females remains unknown and needs to be investigated.

### 3.2 Glucose metabolism

Adiponectin has been shown to participate in the regulation of glucose metabolism (35). As the paralog of adiponectin, CTRP12 also affects glucose metabolism. For instance, decreased serum CTRP12 levels are observed in type 2 diabetes patients as compared to healthy subjects (36). Circulating CTRP12 levels are negatively correlated with insulin resistance in CAD patients (30). Importantly, systemic administration of CTRP12 improves glucose intolerance and inhibits insulin resistance in diet-induced obese mice (14). Further studies showed that CTRP12 augments insulin secretion and increases tyrosine phosphorylation of insulin receptor substrate (IRS-1), leading to reduced blood glucose in leptin-deficient *ob/ob* mice (16). These observations identify CTRP12 as an insulin-sensitizing adipokine. In addition, CTRP12 can directly activate the phosphatidylinositol 3-kinase/Akt axis to antagonize gluconeogenesis and facilitate glucose uptake in diet-induced obese mice, suggesting the involvement of insulin-independent mechanism in glucose-lowering action of CTRP12 (16). Of note, insulin can also up-regulate CTRP12 expression (24). Thus, a positive feedback loop exists between CTRP12 and insulin.

### 3.3 Inflammatory response

In addition to lipid and glucose metabolism, CTRP12 is implicated in inflammatory response. Studies from our group demonstrated that overexpression of CTRP12 promotes the polarization of macrophages to M2 phenotype along with decreased monocyte chemotactic protein-1 (MCP-1) and TNF-α levels as well as increased interleukin (IL)-10 levels (37). Mechanistically, CTRP12 attenuates miR-155-5p levels and then up-regulates its target gene liver X receptor α (LXRα) expression to alleviate inflammatory response (37). In cardiomyocytes challenged with lipopolysaccharide (LPS), CTRP12 inhibits the transcription and subsequent release of TNF-α, IL-1 and IL-6 (38). In addition, serum CTRP12 levels show a negative correlation with TNF-α and IL-6 in CAD patients (30). Taken together, these findings reveal a protective effect of CTRP12 on inflammation.

### 3.4 Oxidative stress

Oxidative stress is defined as the imbalance between the generation of reactive oxygen species (ROS) and the endogenous antioxidant defence system. Excessive oxidative stress can cause protein and lipid peroxidation, impair DNA, and ultimately result in irreversible cell damage and death (39). It has recently been demonstrated that overexpression of CTRP12 markedly reduces cardiac ROS amount in a rat model of post-myocardial infarction heart failure, and an opposite effect appears upon CTRP12 knockdown (40). This effect is mediated by the inactivation of transforming growth factor-β activated kinase 1 (TAK1)-p38 MAPK/c-Jun N-terminal kinase (JNK) pathway (40). In LPS-stimulated cardiomyocytes, CTRP12 decreases ROS and malondialdehyde levels but increases superoxide dismutase activity by up-regulating nuclear factor E2-related factor 2 (Nrf2) expression (41). Another study showed that CTRP12 activates the Nrf2 signaling to alleviate oxidative stress in cardiomyocytes exposed to hypoxia/re-oxygenation (42). Thus, CTRP12 may be a powerful candidate to develop a preventive agent for oxidative stress.

### 3.5 Cardiomyocyte apoptosis

Apoptosis is a type of programmed cell death manner with characteristic morphological and biochemical alterations. Cell apoptosis plays an important role in regulating many physiological and pathological processes (43–45). CTRP12 was found to repress cardiomyocyte apoptosis through the TAK1-p38 MAPK/JNK pathway in rats with post-myocardial infarction heart failure (40). In cardiomyocytes challenged with hypoxia/re-oxygenation, the incidence of apoptosis is significantly decreased in response to CTRP12 (42). In LPS-treated cardiomyocytes, CTRP12 markedly lowers the number of TUNEL-positive cells and augments the ratio of Bcl-1/Bax in a Nrf2-dependent manner (41). In addition, KLF15 reduces cardiomyocyte apoptosis under the condition of hypoxia-reoxygenation in a CTRP12-dependent manner (46). These data provide direct evidence to indicate an anti-apoptotic effect of CTRP12. Oxidative stress is a well-recognized upstream trigger of apoptosis (47). As mentioned above, CTRP12 contributes to the alleviation of oxidative stress. Therefore, CTRP12-mediated regulation of oxidative stress might contribute to its anti-apoptotic effect. Future research is required to confirm this possibility. Of note, adiponectin has been reported to protect endothelial cells and vascular smooth muscle cells (VSMCs) against apoptosis (48, 49). However, the impact of CTRP12 as an important member of the adiponectin family on vascular cell apoptosis is still largely unknown and needs to be investigated.

## 4 Role of CTRP12 in CVDs

**Figure 3:**
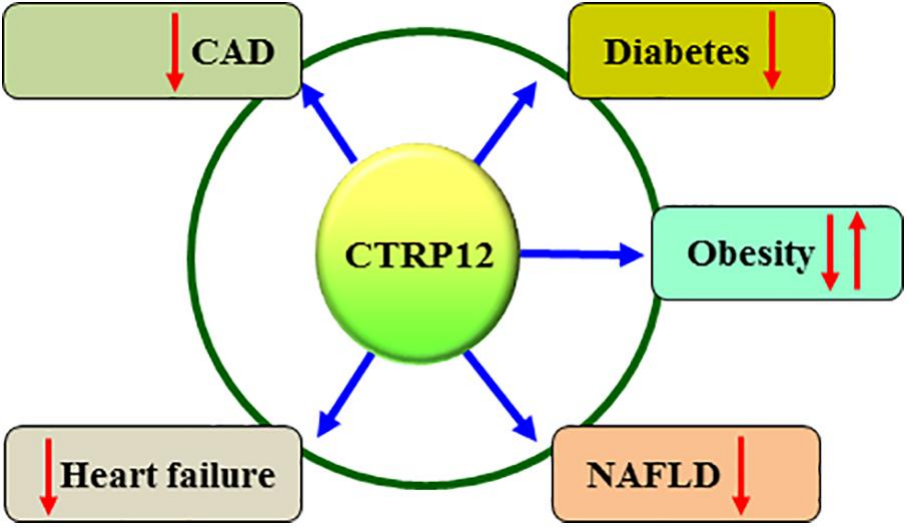
Relevance of CTRP12 to cardiovascular and metabolic diseases. Dysregulation of CTRP12 is closely related to the onset and development of cardiovascular and metabolic diseases. In particular, CTRP12 has a protective effect on CAD, heart failure, diabetes and NAFLD. However, CTRP12 plays a dual role in obesity progression, depending on the gender.

CVDs are a group of disorders affecting heart and blood vessels and cause a significant global health burden. CTRP12 has been shown to be involved in the occurrence and development of CVDs, such as CAD and heart failure (Figure 3 and Table 1).

**Table 1:** Effect of CTRP12 on cardiovascular and metabolic diseases.

Disease types	Molecular axis	Functions	Actions	References
CAD	Nrf2	Inhibiting cardiomyocyte injury	Protection	(41, 42)
	Unknown	Alleviating cardiomyocyte damage	Protection	(46)
	miR-155-5p/LXRα/ABCA1/ABCG1	Inhibiting macrophage foam cell formation	Protection	(31)
	miR-155-5p/LXRα	Promoting M2 macrophage polarization	Protection	(31)
	TGF-βRII/Smad2	Inhibiting inflammatory response and VSMC proliferation	Protection	(70)
	Unknown	Improving plasma lipid profile	Protection	(72)
Heart failure	TAK1-p38 MAPK/JNK	Improving cardiac function, inhibiting cardiac hypertrophy and attenuating cardiac fibrosis	Protection	(40)
	p38	Suppressing extracellular matrix synthesis	Protection	(87)
Diabetes	Unknown	Increasing insulin sensitivity	Protection	(99)
Obesity	Unknown	Reducing body weight and subcutaneous adipose tissue mass	Protection	(104)
NAFLD	GPAT and DGAT	Suppressing triglyceride biosynthesis	Protection	(32)

### 4.1 CAD

CAD is the most common cardiovascular disease constituting a severe threat for human health and consists of stable angina, unstable angina, myocardial infarction and sudden cardiac death (50). In recent years, the association of CTRP12 with CAD has gained a great attention. Serum CTRP12 levels in CAD patients are lower than those in healthy individuals and have an independent association with the risk of CAD (30). Another case-control study showed that serum CTRP12 levels are negatively correlated with the severity of CAD (51). Acute coronary syndromes (ACS) are characterized by a sudden reduction in blood supply to the heart and refers to a spectrum of coronary artery pathologies, including ST-segment elevation myocardial infarction, non-ST-segment elevation myocardial infarction and unstable angina (52–54). As a primary health concern, ACS causes a huge economic burden and accounts for more than 1 million hospitalizations annually in the United States (55). A recent study showed that serum CTRP12 levels are significantly decreased in ACS patients as compared to healthy controls, and elevated CTRP12 levels function as an independent protective factor for ACS (56). These data indicate that circulating CTRP12 may possess enormous potential to act as a novel predictor for cardiovascular risk and disease severity.

Cardiomyocyte injury and death are regarded as a key driver for CAD, especially myocardial infarction (57–59). It has been demonstrated that overexpression of CTRP12 alleviates LPS or hypoxia/reoxygenation-induced cardiomyocyte impairment by inhibiting inflammatory response, oxidative stress and cell apoptosis in a Nrf2-dependent manner (41, 42). In a mouse model of myocardial ischemia-reperfusion, the expression levels of KLF15 and CTRP12 are significantly decreased in myocardium (46). Importantly, KLF15 induces CTRP12 transcription to inhibit inflammation and apoptosis, leading to improvement of hypoxia/reoxygenation-caused cell damage in rat myoblast H9c2 cells (46). Thus, targeting CTRP12 may have enormous potential for the prevention and treatment of CAD.

**Figure 4:**
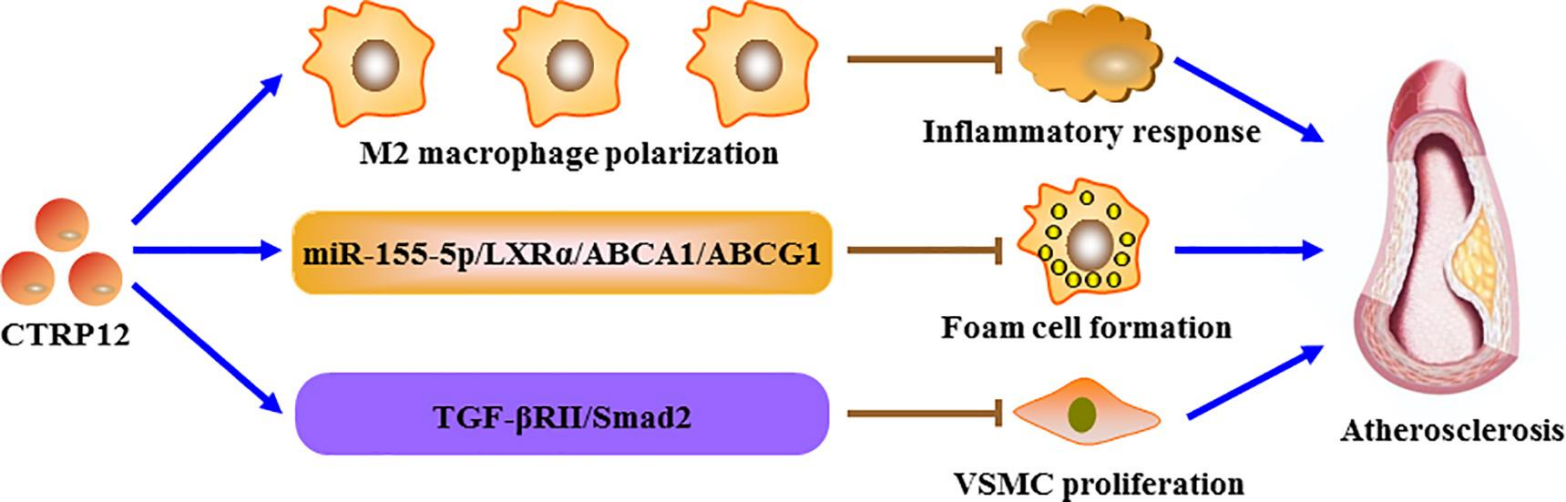
The role of CTRP12 in atherosclerosis. CTRP12 protects against the development of atherosclerosis through three pathways. Firstly, CTRP12 induces the polarization of macrophages toward a M2 phenotype to alleviate inflammatory response. Secondly, CTRP12 up-regulates the expression of ABCA1 and ABCG1 via the miR-155-5p/LXRα signaling cascade, leading to inhibition of macrophage foam cell formation. Thirdly, CTRP12 attenuates VSMC proliferation by activating the TGF-βRII/Smad2 axis.

Atherosclerosis is a lipid-driven inflammatory condition affecting large and medium-sized arteries and functions as the pathological basis of CAD (60). As a major cell type within atherosclerotic plaques, macrophages are implicated in all stages of atherosclerosis progression, including plaque formation, calcification, rupture and regression (61). During atherogenesis, monocytes in the blood stream enter the subendothelial space of arterial intima where they differentiate into macrophages. After uptake of abundant lipids, macrophages are transformed into foam cells, the hallmark of early fatty streak lesions (62–64). ATP binding cassette transporter A1 (ABCA1) and G1 (ABCG1) belong to the integral membrane proteins and play a crucial role in promoting the release of intracellular cholesterol and phospholipids. There is increasing evidence that decrease in ABCA1- and ABCG1-dependent cholesterol efflux is a critical contributor to lipid-laden foam cell formation (65–67). Also, macrophage foam cells can secrete a variety of pro-inflammatory cytokines, leading to aggravation of lipid metabolism disorder and atherosclerotic lesion expansion (68, 69). Our group recently demonstrated that overexpression of CTRP12 inhibits macrophage lipid accumulation and inflammatory to protect against atherosclerosis in mice (31). Further studies revealed that CTRP12 activates the miR-155-5p/LXRα signaling pathway, which enhances ABCA1- and ABCG1-dependent cholesterol efflux to inhibit foam cell formation as well as facilitates M2 macrophage polarization to alleviate inflammation (31). In addition, treatment of mouse peritoneal macrophages with recombinant CTRP12 protein down-regulates the expression of TNF-α, IL-6 and MCP-1, and this beneficial effect is reversed by blocking the transforming growth factor-β receptor II (TGF-βRII)/Smad2 signaling pathway (70). It is well known that low-density lipoprotein cholesterol (LDL-C), total cholesterol and triglycerides are the major risk factors for atherosclerosis development, whereas HDL-C is protective against atherogenesis. A 1 mg/dl increase in plasma HDL-C levels is estimated to diminish the risk of CAD in men by 2% and women by 3% (71). A recent study showed that the combination of spinach thylakoid extract with high-intensity functional training reduces plasma LDL-C, total cholesterol and triglyceride levels but elevates HDL-C levels in obese male subjects, which is possibly attributed to the activation of the KLF15/CTRP12 pathway (72). These data suggest that CTRP12 is an adipokine with atheroprotection (Figure 4), and up-regulation of CTRP12 expression could be a useful therapeutic option for atherosclerosis. Like macrophages, VSMCs can be converted to foam cells after accumulation of large amounts of lipids. It has been demonstrated that approximately 50% of total foam cells are VSMC-derived in human coronary atherosclerotic plaques (73). However, it has not yet been determined whether CTRP12 plays a role in the transformation of VSMCs into foam cells.

Pathological vascular remodeling is a key feature during the progression of various vascular diseases (74, 75). The proliferation and migration of VSMCs is implicated in the pathogenesis of pathological remodeling of the arterial wall, in particular atherosclerosis (76, 77). In mice undergoing wire-induced injury of the femoral artery, deficiency of CTRP12 increases neointimal thickening after vascular injury and proliferative capacity of vascular cells in injured arteries (70). Consistently, CTRP12 significantly decreases platelet-derived growth factor-BB-stimulated proliferation of VSMCs via activation of the TGF-βRII/Smad2 signaling cascade (70). Therefore, prevention of VSMC proliferation is another important mechanism for the atheroprotection of CTRP12. However, the impact of CTRP12 on VSMC migration remains uncompleted and needs to be investigated.

The endothelium, a cellular monolayer located in the blood vessel wall, contributes to the dynamic maintenance of angiogenesis, vascular tone, blood fluidity and permeability. The damage of vascular endothelial cells can result in endothelial dysfunction that is regarded as a critical driver of atherogenesis (78–80). There is increasing evidence that adiponectin is protective against atherosclerosis by alleviating endothelial dysfunction (81, 82). Considering CTRP12 as a paralog of adiponectin, it is highly possible that CTRP12 inhibits the development of atherosclerosis by improving endothelial function. Future studies will be needed to verify this possibility.

### 4.2 Heart failure

Heart failure, which is an endpoint stage of numerous heart diseases, severely jeopardises the health of a large population across the world (83). Cardiac remodelling, including myocardial hypertrophy and fibrosis, plays a critical role in promoting the occurrence and development of heart failure (84, 85). Myocardial infarction is regarded as the most common cause of heart failure (86). A significant reduction of CTRP12 expression is observed in the hearts of rats with post-myocardial infarction heart failure (40). Moreover, overexpression of CTRP12 improves cardiac function, inhibits cardiac hypertrophy and attenuates cardiac fibrosis by inactivating the TAK1-p38 MAPK/JNK pathway in rats with post-myocardial infarction heart failure (40). Another research revealed that in neonatal rat cardiac fibroblasts stimulated with isoproterenol, CTRP12 prevents the transformation of fibroblasts to myofibroblasts and attenuates the synthesis of extracellular matrix by inhibiting the activation of p38 (87). These authors also found that CTRP12 suppresses cardiac fibrosis and enhances cardiac function in mice injected with isoproterenol by attenuating p38 phosphorylation (87). Thus, targeting CTRP12 could be a novel and promising strategy for the management of heart failure.

Heart failure can be classified into three groups based on the percentage of ejection fraction: heart failure with reduced ejection fraction (HFrEF), heart failure with mildly reduced ejection fraction (HFmrEF), and heart failure with preserved ejection fraction (HFpEF) that is defined as heart failure with an ejection fraction of 50% or higher at diagnosis. HFmrEF can progress into either HFrEF or HFpEF, but its phenotype is dominated by CAD, as in HFrEF (88). HFrEF and HFpEF present with differences in both the development and progression of the disease secondary to changes at the cellular and molecular levels. During the last two decades, the incidence and prevalence of HFrEF were decreasing, whereas those of HFpEF continued to rise in tandem with the increasing age and burdens of obesity, sedentariness and cardiometabolic disorders (89). Nevertheless, the role of CTRP12 in HFpEF remains largely unknown. Future studies in this field might be of great scientific significance and clinical value.

## 5 Role of CTRP12 in metabolic diseases

It is well known that metabolic diseases, such as diabetes, obesity and NAFLD, are strongly associated with an increased risk of CVDs (90, 91). Similar to CVDs, CTRP12 is also implicated in the physiopathological processes of these metabolic diseases (Figure 3 and Table 1).

### 5.1 Diabetes

Diabetes is a global disease and has become a public health problem that seriously threatens people's health. Accumulating evidence has demonstrated that adiponectin is closely associated with diabetes (92, 93). Like adiponectin, CTRP12 is involved in the progression of diabetes. The patients with type 2 diabetes mellitus exhibit a marked reduction in serum CTRP12 levels (94). Similar to this report, Du et al. collected 115 type 2 diabetes mellitus patients and 54 healthy subjects and found that serum CTRP12 levels are significantly decreased in diabetic patients compared with healthy controls (36). Polycystic ovary syndrome is regarded as a pro-inflammatory state associated with diabetes. There is a negative correlation between serum CTRP12 concentration and blood glucose levels in women with polycystic ovary syndrome (25, 95). Diabetic nephropathy, a common microvascular complication with a high incidence in diabetic patients, is the major reason for the elevation in mortality of patients (96, 97). These authors also demonstrated that serum CTRP12 levels in diabetic nephropathy patients are lower than those in diabetic patients without diabetic nephropathy and show a negative correlation with diabetes duration, blood urea nitrogen, uric acid and 24-h urinary albumin excretion rate (36). These data suggest that circulating CTRP12 acts as a novel and valuable biomarker for the prediction of renal dysfunction in type 2 diabetes mellitus.

Insulin resistance is a critical feature of diabetes. Treatment with rosiglitazone, an insulin-sensitizing drug, markedly enhances CTRP12 mRNA amount in adipocytes; however, both the mRNA and serum levels of CTRP12 are significantly decreased in insulin-resistant obese (*ob/ob*) mice (16). An inverse correlation between serum CTRP12 levels and insulin resistance is found in CAD patients (30). Thromboxane A2 (TXA2) is an arachidonic acid-derived eicosanoid synthesized by thromboxane synthase (TBXAS). TXA2 signals through its receptor (TBXA2R) and plays a key role in haemostasis (98). In genetic and dietary mouse models of obesity and diabetes, the expression levels of TBXAS and TBXA2R are significantly increased in adipose tissue (99). Deficiency of TBXAS enhances insulin sensitivity and improves glucose homeostasis in mice fed a low-fat diet, which is correlated with incremental CTRP12 expression (99). Under the condition of high-fat diet, knockout of CTRP12 in male mice leads to a significant increase in fasting blood glucose levels, whereas this indicator is not different between female wild-type and CTRP12-deficient mice, suggesting a sex-dependent effect of CTRP12 on insulin sensitivity (100). Dietary intervention in combination with insulin aspart reduces 2-h postprandial blood glucose levels and improves clinical outcomes in pregnant women with gestational diabetes mellitus, which is partially attributed to increased CTRP12 secretion (101). Collectively, these data indicate that CTRP12 is an insulin-sensitizing adipokine and represents a novel target molecule for the treatment of insulin resistance and diabetes.

### 5.2 Obesity

Obesity is a well-known metabolic disease. In recent years, obesity and its associated health issues have risen dramatically and become a global challenge. When compared with the normal weight women, the expression levels of CTRP12 mRNA are significantly increased in subcutaneous adipose tissue and visceral adipose tissue isolated from obese women undergoing bariatric surgery (102). Concurrently, there is a significant correlation between CTRP12 expression and obese indices including waist circumference, hip circumference and body mass index (102). Another research showed that diet-induced obese mice have lower plasma CTRP12 levels (27). However, no significant alterations are observed in serum CTRP12 levels between overweight/obese and normal weight subgroups in polycystic ovary syndrome and non-polycystic ovary syndrome women (103). These controversial results suggest whether circulating CTRP12 can serve as a valuable biomarker of obesity needs further investigation. In obese male Wistar rats induced by high-fat diet, aerobic exercise combined with supplementation of the hydroalcoholic extract of Rosa canina fruit seed can effectively reduce body weight and subcutaneous adipose tissue mass by up-regulating irisin and CTRP12 expression (104). When challenged with a high-fat diet, knockout of CTRP12 in male mice leads to a significant increase in weight gain and adiposity, whereas female CTRP12-deficient mice have reduced weight gain (100). These findings reveal a sex-dependent effect of CTRP12 on obesity.

### 5.3 NAFLD

NAFLD is defined as the presence of steatosis in more than 5% of liver cells with little or no alcohol consumption (105). It contains the benign non-alcoholic fatty liver, and the more severe non-alcoholic steatohepatitis characterized by steatosis, hepatocellular ballooning, lobular inflammation and almost always fibrosis (106–108). Due to the rising prevalence of obesity and type 2 diabetes mellitus, NAFLD is becoming the major cause of chronic liver disease worldwide and a growing challenge for public health. Give the important regulatory role of CTRP12 in lipid metabolism and adiposity, it is not surprising that this adipokine is involved in the onset and development of NAFLD. In hepatoma cells and primary mouse hepatocytes, CTRP12 was shown to suppress triglyceride biosynthesis by down-regulating GPAT and DGAT expression (32). In male mice fed a high-fat diet, partial deficiency of CTRP12 leads to more lipid accumulation and greater steatosis in the liver compared with wild-type littermates (33). Therefore, CTRP12 exerts a protective effect on NAFLD, and promoting CTRP12 synthesis could be an effective strategy for reducing NAFLD.

## 6 Conclusion and future directions

CTRP12 is a newly discovered adipokine and plays an important role in regulating glucose and lipid metabolism, inflammatory response, oxidative stress and cardiomyocyte apoptosis. Importantly, this adipokine has been shown to protect against the occurrence and development of several major cardiovascular and metabolic diseases, including CAD, heart failure, diabetes, obesity and NAFLD. Thus, exogenous administration of CTRP12 or promoting its endogenous production could have considerable promise in the management of these diseases.

Despite significant progress has been made in understanding the role of CTRP12 in the pathogenesis of cardiovascular and metabolic diseases, some concerns still need to be addressed in future studies. CTRP12 is a secreted protein and its receptor is unclear to date. Adiponectin is known to signal through three receptors: T-cadherin, adiponectin receptor 1 (AdipoR1) and AdipoR2 (109). As a G-protein-coupled seven-transmembrane protein, AdipoR1 also acts as the receptor of CTRP1 and CTRP9 (110, 111). Whether this protein is a receptor of CTRP12 needs further investigation. Identification of the specific receptors of CTRP12 will help deeper clarify its mechanisms of action. Given the fact that most of the evidence is limited to the findings of lower plasma CTRP12 concentration in patients as compare to healthy control, more mechanistic studies should be performed to clearly reveal a causal relationship between CTRP12 and cardiovascular and metabolic diseases. Hypertension is a risk factor for early CVDs and is a growing public health problem worldwide. Certain members of CTRPs, such as CTRP1, CTRP3 and CTRP9, have been shown to be associated with hypertension (112–115). It is unclear, however, whether CTRP12 is involved in the development of hypertension. Similar to cell apoptosis, cell pyroptosis is a programmed cell death manner characterized by inflammasome activation and abundant release of pro-inflammatory cytokines (116). Ferroptosis is another type of regulated cell death resulting from lipid peroxidation and iron overload (117, 118). Accumulating evidence has indicated a close association of cell pyroptosis and ferroptosis with the progression of cardiovascular and metabolic diseases (119–122). Nevertheless, the influence of CTRP12 on cell pyroptosis and ferroptosis remains poorly understood and are required to be investigated. Extensive data have demonstrated that several conserved lysine residues in the collagenous domain of adiponectin can be modified by glycosylation and hydroxylation (123, 124). Considering CTRP12 as a paralog of adiponectin, future research should explore the impact of post-translational modifications on CTRP12 structure and functions. To the best of our knowledge, there are no adipokine-targeted drugs available for clinical applications to date. Therefore, development of the drugs targeting adipokines, such as CTRP12, is important to prevent and treat cardiovascular and metabolic diseases. In addition, sterol regulatory element-binding protein 2 and 3-hydroxy-3-methylglutaryl-CoA reductase are known to play a crucial role in promoting endogenous cholesterol biosynthesis. Aberrant expression of these two agents is correlated with lipid metabolism disorder and atherogenesis (125–127). To establish further link of CTRP12 to lipid metabolism, it is of great importance to investigate the regulatory effect of CTRP12 on their expression. In summary, CTRP12 is multifunctional adipokine and an emerging player in cardiovascular and metabolic diseases. A more comprehensive understanding of the relationship of CTRP12 with cardiovascular and metabolic diseases will shed light on the development of CTRP12-targeted treatment.
